# Social representations of cancer in patients from Medellín, Colombia: a qualitative study

**DOI:** 10.3389/fsoc.2023.1257776

**Published:** 2023-12-01

**Authors:** Luis Felipe Higuita-Gutiérrez, Diego Alejandro Estrada-Mesa, Jaiberth Antonio Cardona-Arias

**Affiliations:** ^1^School of Medicine, Universidad Cooperativa de Colombia, Medellín, Colombia; ^2^School of Microbiology, Universidad de Antioquia, Medellín, Colombia

**Keywords:** cancer, social representations, grounded theory, ideology of positive thinking, medical sociology

## Abstract

**Background:**

Cancer has different explanatory theories that address its etiology and treatment. It is usually associated with pain and suffering. Recently, new technologies, knowledge, and therapies have been developed, which may have transformed the classic social representations of the disease. This study aimed to understand the social representations (SRs) of cancer in patients from Medellín, Colombia.

**Methods:**

This study used a grounded theory in 16 patients with cancer. The information was collected between June 2020 and May 2021. Information was analyzed following the open, axial, and selective coding stages.

**Results:**

SRs of cancer at the time of diagnosis evoke negative connotations. However, cancer is redefined as a positive event as the clinical course of the disease progresses, and patients interact with health professionals and respond to treatment. The resignification of the disease depends on the etiological models of the patients, which include genetic, socio-anthropological, psychosocial, and psychogenic factors. In line with the SRs of etiology, patients seek out treatments complementary to the biomedical ones that can be socio-anthropological and psychogenic.

**Conclusion:**

In this group negative representations about cancer persist, this way of understanding the disease is determined by the convergence of cultural meanings and personal experiences. The causal representation is connected to the actions and willingness of the patients to face their diagnosis. In this sense, two categories stand out: the first expresses that cancer is the consequence of a body subjected to excessive productivity; the second subsumes a psychogenic predisposition caused by the context where the ideology of happiness appears to be a social norm. This double saturation in which an individual is immersed results in new burdens that are not visible to caregivers and healthcare workers.

## 1 Introduction

Cancer is one of the most important barriers to globally increasing life expectancy, with approximately 10 million deaths and 19.3 million new cases in 2020 (Sung et al., 2021). Its lethality, treatment complexity, and fear of death have contributed to this pathology being socially feared. This has been related to different explanatory theories about the origin of cancer in different historical periods (Hajdu, 2016).

In ancient times, the intervention of evil spirits, supernatural forces, and planetary movements were thought to be causative factors of cancer. In Greece and Rome, etiology was linked to sin and the wrath of the gods. Hippocrates suggested that the cause was an excess or deficiency of blood, mucus, and bile. Galen ascribed it to the accumulation of black bile (Pearson, 1793). In the Middle Ages, its origin was attributed to the accumulation of harmful substances in the blood. At the end of the 18th-century, the cancerous diathesis indicated that trauma, irritation, and inflammation, in addition to certain people’s predisposition, caused the disease (Pearson, 1793). Subsequently, hereditary predisposition was added. In the 19th-century, Snow (1793) linked the disease to mental and emotional disturbances, and intense suffering. Nowadays, the International Agency for Research on Cancer has described about 500 agents with the potential to cause the disease; these are considered necessary but not sufficient causes, given that cancer is caused by the convergence of environmental pollutants, diet, obesity, physical inactivity, smoking, alcoholism, hormonal factors, microbial agents, heredity, occupation, and others (International agency for research on cancer, 2022).

The plurality of explanatory cancer theories coincides with the heterogeneity of its treatments, which include: spiritual, mystical, and religious therapies; surgeries, chemotherapy, radiotherapy, immunotherapy, gene therapy, psychotherapy, medicinal plants, acupuncture, mindfulness, and others (Arruebo et al., 2011; Lee et al., 2019).

The consequences of the disease, diversity of explanatory theories, multiplicity of treatments, and socioeconomic and cultural conditions have a direct impact on the social representations (SR) of cancer in patients. SRs refer to socially elaborated knowledge built from experiences, information, and models of thought received and transmitted by education, tradition, and social communication (Araya, 2002). SRs of a disease are nourished by the information that patients receive from social contacts, previous cultural knowledge, their relationship with physicians and medical institutions, and their own experience with the disease. The interest in SRs is because they are related to patients’ discourse and practices; thus, they are linked to help-seeking at the onset of symptoms, early diagnosis, selection of a type of treatment, and therapeutic adherence (Araya, 2002; Canelón et al., 2003).

Different studies in the literature have accounted for the SR of cancer in general (Wakiuchi et al., 2020), prostate, head, neck, and breast cancer (Hébert et al., 2016; Formigosa et al., 2018), or their treatments (Marie et al., 2010; Albarghouthi and Klempe, 2019). In Colombia, SRs of cancer have been studied in Bogotá (Palacios and Zani, 2014). Furthermore, a study on the SRs of breast cancer in Medellín (Giraldo-Mora, 2009), and a doctoral thesis on the SRs of cervical cancer in the indigenous Wuayu population (Cortés, 2016) have been published. These Colombian studies have revealed that the SRs of cancer and chemotherapy continue to be negative, and are associated with words such as death, terror, suffering, and pain, which explain the anxiety and suffering that is generated at the time of diagnosis.

In 1988, Susan Sontag wrote that cancer had ceased to be a stigma that damaged identity because, by that time, people talked about cancer more freely with less secrecy, patients were more informed, and doctors avoided hiding the diagnosis for fear of litigation (Sontag, 2003). In addition, the treatment has made substantial progress, such as more precise and minimally invasive surgeries, better chemotherapies, more precise radiotherapies, and molecular characterizations that allow for targeted therapies and immunotherapies, increasing the 5 years survival rate in more than 90% of patients with some types of cancer (Advancing Cancer Therapy, 2021). Because of this, the prognosis and quality of life have substantially improved.

As recent studies have suggested, these events are producing new cancer SRs (Pocinho et al., 2021). In other pathologies, Christiane (2006) pointed out that the development of new technologies, knowledge, and therapies impact the ideas about disease, and the values and practices of patients with respect to medical norms and their lifestyles. This study is part of a new initiative that explore the changes in cancer SRs because of technological, social, and cultural transformations in recent decades. In view of the above, this study was designed with the objective of to understand the SRs of cancer, its etiology, and treatment in patients from Medellín-Colombia.

## 2 Methods

### 2.1 Type of study

Grounded theory in which SRs were studied from a processual approach, characterized by privileging the analysis of culture, and social interactions (as opposed to the structural approach which cognitive and psychic dimension predominates). The postulates of symbolic interactionism were followed. This perspective states that people act based on the meanings of phenomena; these meanings emerge from social interaction and are transformed by the interpretations that people make of these phenomena (Blumer, 1993). To these meanings (symbolic or hermeneutic reality), the SRs theory adds socio-structural aspects and material frameworks (social reality) where people live, which define their understanding of reality to some extent (Araya, 2002).

### 2.2 Study subjects

The study included 16 patients with cancer over 18 years of age whose disease was diagnosed ≤2 years prior and who were treated in Medellín, Colombia. Sampling was performed using the snowball technique, considering the criteria of maximum variability for age (22 to 71 years), social class, educational level (primary school to postgraduate), and type of cancer (thyroid, breast, stomach, prostate, cervix, leukemia, and lymphoma).

### 2.3 Information collection

Data were collected between June 2020 and May 2021 by tele-assisted interviews because national authorities mandated social distancing to protect patients and researchers from infection by the SARS-CoV-2 virus. The interviews contained pre-established questions about the words and emotions that the term “cancer” evoked, the aspects to which the patients attributed the disease, and the treatments that they underwent. These questions were complemented with spontaneous interventions by the researchers according to the flow of the conversation, thus fulfilling the criteria of semi-structured interviews. Each patient underwent two to three interviews consistent with the open, axial, and selective coding stages of grounded theory (Strauss and Corbin, 2012).

### 2.4 Information analysis

The first step consisted of familiarizing the researchers with the interviews. The recordings were repeatedly listened, and transcribed for later coding and categorization. During open coding, texts were broken down and closely examined for fragments conceptually similar or related in meaning. These fragments were assigned a label, either based on the words of the patients (*in vivo* code) or concepts from the literature (pre-established code). Labels were grouped into categories from a higher-order abstract. In axial coding, the data of open coding were regrouped, and the concepts and categories were linked with subcategories. In selective coding, a larger theoretical scheme was formulated, and the significance matrix was built based on the choice of a core category and its relationship with the others. Finally, the theoretical framework was validated by cross-checking it with the data and interviewees (Strauss and Corbin, 2012).

### 2.5 Quality criteria

(i) Theoretical and inter-researcher triangulation, (ii) validation by the respondent to corroborate that the researchers’ interpretations coincided with what the patients wanted to express, (iii) reflexivity with a field diary during data collection and analysis, and (iv) inclusion of various perspectives with maximum variation sampling (Pope and Mays, 2020).

### 2.6 Ethical considerations

All participants recorded their informed consent, which reported the objectives, procedures, risks, benefits, and voluntariness of the study. The procedures were approved by the bioethics committee of the Universidad Cooperativa de Colombia record 027-2020 min 005. This study was conducted according to the guidelines of the Declaration of Helsinki, and Resolution 8,430 of the Ministry of Health of Colombia of 1993.

## 3 Results

The age of the 16 patients ranged between 22 and 71 years, came from all social classes (low to high), and had educational levels ranging from elementary school to postgraduate studies (five healthcare professionals). The types of cancer were stomach, prostate, cervical, leukemia, lymphoma, thyroid, and breast (Table 1).

**Table 1:** Description of participants’ characteristics.

Sex	Age (years)	Type of cancer	Occupation	Monthly income (COP)	Social class
Male	23	Lymphoma	Unemployed	$0	Low
Female	53	Breast	Housewife	$500.000	Low
Female	33	Thyroid	Secretary	$1.100.000	Low
Female	52	Breast	Housewife	$850.000	Low
Female	53	Cérvix	Businesswoman	$0	Low
Male	22	Stomach	Univ. student	$700.000	Middle
Female	51	Breast	Dental assistant	$1.800.000	Middle
Female	55	Breast	Housewife	$2.500.000	Middle
Female	71	Breast	Secretary	$1.500.000	Middle
Female	26	Thyroid	Physician	$2.700.000	High
Female	27	Leukemia	Physician	$8.000000	High
Female	29	Leukemia	Physician	$3.900.000	High
Female	58	Thyroid	Bacteriologist	–	High
Female	61	Leukemia	Retired	$2.000.000	High
Male	61	Prostate	Odontologist	$3.000.000	High
Male	64	Prostate	Public accountant	$10.000.000	High

### 3.1 Representations of cancer: expression of negative and positive meanings

At the time of diagnosis, all participants described cancer as a negative event associated with pain, suffering, hospitalization, treatment and death. Some patients were reluctant to speak explicitly about cancer. They withhold the name of the disease because of the shock that the word generates.

“Cancer is… ohhh no, it's a horrible word. One always associates it with bad things. I associate it with illness, death, treatment and hospitalization”. **Woman, 37 years old, with leukemia.**

“One even tries to avoid mentioning it because one always associates cancer with death. I don’t think there is anyone who hears it and remains very calm”. **Man, 64 years old, prostate cancer.**

The “cancer = death” equation was the most recurrent representation. This way of understanding the disease is embedded in the culture. This representation is permanently circulated and mobilized by individuals, and exerts a strong influence on their belief system to the point of resisting scientific rationality and medical knowledge. A physician with cancer said the following:

“To me, the world was shut, and I didn't think like a doctor, but I thought ‘oh no, I have cancer, I'm going to die.’ I believe that there is a concept within the population that associates the word ‘cancer’ with death. So, I believe it is something that they have taught us. Even before becoming a doctor, they tell you that cancer equals death. It is more about what you have been taught throughout your life.” **Woman, 29 years old, with lymphoma.**

On the other hand, the largest group of patients (heterogeneous by schooling, social class, type of cancer, and knowledge of health sciences) reported positive meanings; they had a resignification of the disease when they began to receive health care and responded to treatment. They begin to understand the body as a battlefield in which there is an enemy to be defeated (assuming a fighting attitude). Now, cancer evokes positive aspects such as change, transformation, proof, opportunity, and is an important source of life lessons. The disease edified them as people; it divided their lives into two, and allowed them to place value on time, family, and emotions.

“I see it as if I was one person before and another after. In my case, the wound was very large and was a physical wound, unspiritual or emotional; however, I feel that the wound united my heart and head. I was thinking, thinking, thinking… with the wound, those two centers (heart and head) were united. The other thing is that I feel that acceptance, compassion, and love came through the wound, and that made me a better person. A better, different, and considerably improved person. So I say welcome. I'm not wishing for cancer, but it depends on how you take it; it has been a wonderful experience for me.” **Woman, 58 years old, with thyroid cancer.**

### 3.2 Representations of the etiology of cancer: genetic, socio-anthropological, psychosocial, and psychogenic factors

For subjects aged over 60 years with no prior knowledge of health sciences, and those who established a subordinate relationship with their physicians, link the etiology of the disease to genetic aspects, which are presented as a unicausal etiology. Genes are the central unit of analysis for cancer, and they are considered to be non-susceptible to individual influence or control; therefore, patients with cancer believe that they are resigned to their fate.

“The disease can come from genes and can be a modification in the genes. Let's say that it is not by doing or not doing an activity that this type of disease occurs. It is not caused by something that I would have done or not done. It is like a lottery that some we win.” **Man, 64 years old, with prostate cancer.**

Other group of patients agreed that the cancer originated from a convergence of social, anthropological, psychological, and biological factors, although one of them predominantly stood out. In the socio-anthropological aspects, some place the disease in a mystical realm (divine design), but the most common account revolves around cultural, ideological, and economic issues that, in their view, have transformed people. In this sense, the disease is attributed to exhaustion derived from the excess of activities, the dilution of the person undertaking multiple tasks (work, study, and social life), the advent of multitasking, internal pressures to maintain high levels of performance, and the exploitation of oneself to the point of no longer being able to cope (*animal laborans*). Although this type of participant (who are different from the health professionals) does not explicitly allude to any scientific basis to connect the stress of the modern-contemporary lifestyle with the cause of cancer, in the explanatory models of the disease from *doxa*-domain (popular knowledge) they indicate the connection of overloaded lifestyles without time for leisure with occurrence of the disease.

“I would get to a point where I would say I can't, I don't have it in me, but [my own] answer was that it was an obligation, it's the responsibility we have as a family. So I had to accomplish several things, and I didn't stop it along the way. From wanting to have a career at the perfect time, perfectly within the syllabus, wanting to work, wanting to have a social life, wanting to exercise. Then you start to force yourself to do lots of things… If you ask me what the specific cause is, I wouldn't know how to tell you, but I feel that all these situations had a lot to do with it; they added up to a lot because that's where you start to lose yourself.” **Man, 22 years old, with stomach cancer**.

Another socioanthropological factor that participants link to the cause of cancer is the external pressure from their work environment to increase productivity. This fact is particularly evident among healthcare professionals who report that their life is reduced to work, and that there is no time for rest, leisure, or food. Thus, work subjects them to conditions that push them to the limit, and from their perspective, this leads to cancer. It is important to highlight that this situation generates cognitive tension among the physicians interviewed: their biomedical knowledge does not recognize or admit their system of beliefs about the etiology of the disease, qualifying it as “fictional,” “irrational,” and “illogical”; even so, the origin of the cancer is attributed to this system.

“I think that having a career in medicine indicates that one does not have as much self-care time as one would think; that is, physicians are always thinking about the other person and rarely think about themselves. You could be working a night shift and not sleeping much, but it doesn't matter. It's 3 p.m. and you've had such a horrible ER shift that you haven't been able to have lunch, but it doesn't matter. You're tired, maybe depressed, and it doesn't matter. I don't have time to exercise, well, it doesn’t matter… If I were to give any cause, however illogical it may be, that would be it.” **Woman, 26 years old, with thyroid cancer.**

Psychogenic factors stated that cancer arises from situations that generate intense suffering, such as death, divorce, pressing economic issues, or sudden changes in social status. This concept emerges from internal dialog and interaction with specialized professionals.

“I believe cancer is an emotional disease because, in 2019, I suffered so much. I believe that I had never suffered how I suffered that year, so my conclusion is that cancer is an emotional disease.” **Woman, 71 years old, with breast cancer**.

“When I was hospitalized, the hematologist told me ‘I'm going to ask you to speak with a psychologist, not because you're sick, but because sometimes we have to talk about the things that we associate with this type of disease.’ I told her I was in great agreement. And speaking with the psychologist, she guided me to think that there could be an association between that emotional part that I had so strongly linked with cancer, because it coincided with that time.” **Woman, 29 years old, with lymphoma.**

In that same sense, the notion of “proto-disease” emerged to refer to the knowledge of individual susceptibility to cancer as a trigger for psychosomatic processes that lead to disease. These accounts mention that the fact that the doctor communicated the risk of getting sick engraved in them a system of diagnostic procedures, medical consultations, and treatments that predisposed them to cancer through psychosomatic mechanisms of thoughts, beliefs, and fears that generated negative expectations about their health, which were strong enough to induce the disease.

“The internist always told me that ‘as your mother and your sister had it [cancer], it's most likely that you will get too.’ So I think that you are predisposed. I believe that sometimes with a belief you can create that reality. So yes, in my case it's difficult to say because there are many things in there anyway. However, I reiterate that, in my case, there may have been a predisposition. I have a proof and it's my older sister. My older sister has worked to say no, it's not going to happen to me, and she's doing great. I think that yes, you can predispose yourself. I don't know what happens for the rest of people, but in my case yes, yes, yes. Totally, totally.” **Woman, 58 years old, with thyroid cancer**.

### 3.3 Representations of cancer treatment: biomedical, socio-anthropological, and psychological treatments

Subjects who link the etiology of the disease to genetic aspects, treat their disease according to biomedical therapeutic recommendations, and undergo surgery, chemotherapy, and/or radiotherapy when required. This group of patients omits the biological relationship with complex social and environmental processes from their narratives; thus, the genetization of the disease erases the context in which the patients became ill.

In relation to treatment, the other groups of patients also adhere to the therapeutic recommendations of biomedicine; however, they mention complementary therapeutic practices that are consistent with the aspects to which they attribute the disease. Thus, within the socio-anthropological factors, stories emerged in which the positive response to treatment was attributed to divine intervention or to a combination of pseudotherapies. The reasons behind these treatments include that the medical institutions expressed limitations in addressing their cancer, they had persistent symptoms, or the improvement did not meet their expectations.

“I am certain in terms of a non-scientific but spiritual certainty that there was a miracle. When I say this, it is because, as I told you, science was done with me, and it gave me days, it counted the days and months – being very optimistic – that I was going to live. So when I mention it that way, it's because there was something beyond that that highlighted that it was not my time. Simply that it was part of a very complex learning experience, with many implications, but that there was a much larger intervention than just a drug”. **Man, 22 years old, with stomach cancer.**

“You look for other alternatives when you see that the medical department isn't giving you another option. If you don't want to die, you try and look elsewhere. One time, a guy told me to try this drink, and I started drinking it. It's hot water with bicarbonate soda, which is excellent at killing cancer cells, and the truth is that I started drinking it and since I started that, I've noticed a lot of improvement.” **Man, 23 years old, with lymphoma.**

Among those who mention that the etiology of the disease is linked to exhaustion generated by their transformation into a performance machine, complementary treatment consists of reducing the number, form, and intensity of activities they perform in their daily lives, as well as changing their work environment. They stress the importance of achieving a balance between the time they dedicate to rest, food, exercise, and work, as well as the need to reduce or eliminate factors that generate stress.

“Well, I started with several situations. I changed jobs. My workload dropped by 60%. Changing jobs and improving relationships resulted in a significant change. Before, I worked very far and had to get up very early to be at the office on time. I worked both in the office and at home, so there was a lot of stress. Then I moved on to another job doing the same thing, but with a better work environment, a better boss, and better co-workers, and it was close to my home; I could come home for lunch. The situation totally changed and my quality of life improved. And I told my children: if I had stayed in the place that I worked at before, I would not have overcome the cancer, and it would have progressed further.” **Woman, 61 years old, with leukemia.**

Finally, the group of patients who linked their disease to psychogenic factors sought characterological treatments, and those who thought of themselves as introspective sought to learn to express themselves more easily. Likewise, those who had feelings related to sadness, fear, and depression tried to cancel them out and engage in activities that led to positive thoughts. In this context, “self-healing” emerges to refer to the discourses of patients who affirm that meditation techniques, positive thoughts, and energies influence cell biology and, consequently, disease course.

“This disease feeds on people's state of mind. If you're calm, serene, and not sad and bored, saying: ‘I have cancer, I have cancer,’ you're going to get better faster than the person who is saying I'm going to die. So I tried to distract my mind a lot, do crossword puzzles, and do the things that would keep my head calm and make me feel cheerful and smile.” **Woman, 52 years old, with breast cancer.**

“It's the ability to think that I have all this capacity to be able to heal myself, that I can achieve it, and that I can heal myself. Every day I perform healing meditations, and I'm sending body positive energies to the cells. I'm in that process. That's why I say that it's a permanent healing process because every day we have to heal ourselves.” **Woman, 58 years old, with thyroid cancer.**

**Figure 1:**
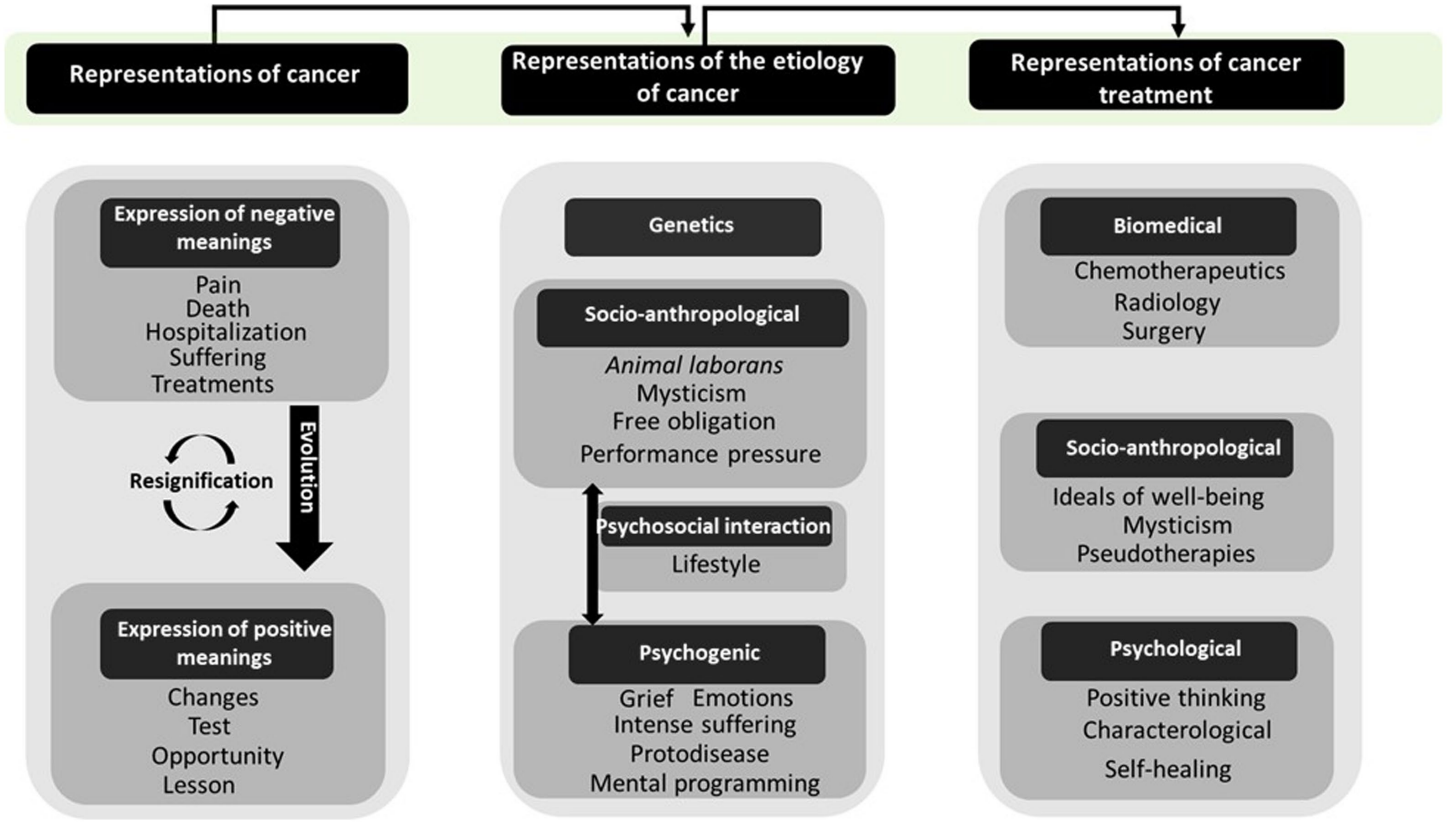
Schematic representation of categories related to the disease, its etiology and treatment.

Based on the above, a significance matrix was built (Figure 1); it shows that the SRs of cancer at the time of diagnosis evoke words with negative connotations. However, cancer is redefined as a positive event in life as the clinical course of the disease progresses, and as patients interact with health professionals and respond to treatment. The resignification of the disease depends on the etiological aspects with which patients link it, and these aspects can be genetic, socio-anthropological, psychosocial, and psychogenic. In line with the SRs of etiology, patients seek out treatments complementary to the biomedical ones; these treatments can be socio-anthropological and psychogenic.

## 4 Discussion

At the time of diagnosis, all participants agreed to describe cancer as a negative event associated with death. This finding coincides with the results of other studies in which death is at the central core of cancer representations (Leitão et al., 2013; Rodríguez and Palácios-Espinosa, 2013; Palacios and Zani, 2014; Wakiuchi et al., 2020); this suggests that this way of understanding the disease is determined by the convergence of cultural meanings and personal experiences. This hypothesis is reinforced by two facts: (i) the fear of the disease is present in diagnosed people but is also prevalent in the general population (Perretti-Watel et al., 2012) and (ii) the “cancer = death” equation resists scientific rationality and the official language of medicine, even in the case of the health professionals included in this study.

The post-diagnosis labeling phenomenon highlights two very different positions that, in turn, have a substantial impact on the field of therapeutics and the “management” of the pathology by patients. As the results show, one group of participants subscribed to a unicausal etiology based on genetic lottery, a materialistic conception of the origin of the disease in which only biomedical discourse has a place. This representation, in turn, explains the ideology of active medicine and a body that is passive when facing medical power; this idea was developed by the Scottish physician John Brown (1735–1788) and set out by Canguilhem (1988). On the other hand, the representations in which the participants adhere to more complex or multicausal conceptions are related to the stances in which the patient assumes a much more protagonist role both in the therapeutic sphere, and in the management of their pathology. In fact, these two positions or perceptions that allude to the origin of cancer, open two practical stances or two types of subjective points of view regarding medical norms.

The results highlight that the group of patients who alluded to the idea of a complex origin of the disease sees the body as a battlefield, and emphasize the importance of assuming a fighting attitude toward cancer. This finding is a reminder that the war metaphor has been present in everything surrounding this disease: cancer cells invade organs, chemotherapy is intended to kill those cells, and the defenses of the body are weakened. The World Health Organization itself generates guidelines for cancer-fighting programs, in which it labels the disease as a silent killer that surreptitiously invades us (World Health Organization, 2002). These types of metaphors have been discussed at length by Susan Sontag in her well-known book, Illness as Metaphor (Sontag, 2003), but the spread of military rhetoric in medicine has been documented since the 17th century (Nie et al., 2016b). Since then, wars against tuberculosis (Lerner, 1993), AIDS (Ross, 1989), diabetes (Safran and Vinicor, 1999), and cancer (World Health Organization, 2002) have been declared. The impact of the use of these metaphors has been studied by medical humanities and cognitive sciences, and the results reveal that they influence patients’ representation of the disease, their judgment, and their decision-making process (Hauser and Schwarz, 2016). Linking a disease, such as cancer, with warlike language decreases people’s intentions to turn away from behaviors that increase the risk of the disease because it highlights aggression rather than self-control (Hauser and Schwarz, 2016). In addition, it has also been found that patients who see themselves as fighters may suffer a loss of self-esteem and a sense of personal failure when they see that their efforts to fight the disease have not been enough to control it (Greer et al., 1979; Malm, 2016). According to a systematic people with cancer should not feel pressured to adopt a fighting spirit to improve their survival or reduce the risk of relapse (Petticrew et al., 2002). Other authors advocate complementary metaphors such as the journey or plural metaphor (Nie et al., 2016a).

Another relevant finding lies in the patients’ testimonies that indicate a change in the representations of the patients’ perceptions regarding disease from the moment they begin to receive care and respond to treatment and begin seeing it as an opportunity for personal growth and an event that has led to positive transformations in different areas of their lives. The fact that an individual may experience better interpersonal relationships, deeper spirituality, or greater inner strength after having experienced a serious illness, such as cancer, has been conceptualized as post-traumatic growth by a current study on positive psychology (Coyne and Tennen, 2010). Publications that refer to this subject have been abundant in scientific literature over the last 20 years (Marziliano et al., 2020) and have given rise to grandiloquent titles in self-help literature, such as “The gift of cancer” (Michaels and Mercant, 2014). In both cases, a significant effect on the well-being of patients is suggested. However, this effect has been measured with scales such as the Post-Traumatic Growth Inventory, which has been the subject of much criticism (Tedeschi and Calhoun, 1996; Coyne and Tennen, 2010). For this reason, it has been argued that contrary to increasing well-being, people who see cancer as something positive end up having poorer mental health (Andrade, 2019). In the same sense, some authors have described that finding benefits in a disease of this type may be another case of the “positive illusions” that characterize human behavior (Taylor and Brown, 1988). Thus, it is necessary to rigorously study this concept because the dissemination of unfounded ideas could have negative effects on patients, such as interfering with the search for help, generating false expectations, and experiencing a higher level of pressure because they do not only need to survive the disease but also to grow and change their identity (Sumalla et al., 2009).

Hacking (1995), philosopher of science, has offered an interpretation that partially explains these findings. He starts from a “nominalist” position, pointing out that an important characteristic of human beings consists precisely of labeling not only natural events but also themselves. However, the images, narratives, or representations that describe or signify “the human” modify the agents by interacting with them. Hacking uses the “looping effect” concept to describe this process, and it can be used in multiple domains. It functions to refer to the multiple labels that serve to frame or classify people, from popular notions to far more formalized concepts, such as those from social or health sciences. However, Hacking emphasizes that language or words alone are not sufficient to bring about any modification in behavior, thoughts, or emotions. It is the interactions between the agents, the words, and the media and institutions that “reinforce,” so to speak, the “looping effects.” Subjection to certain words and interaction with certain metaphors within specific institutional frameworks produce “classes of people.” The results of this study are an example of how the “cancer” label and the production of the “cancer patient” generate heterogeneous responses that determine the norms of life. This means that the diagnosis not only describes an organic reality of the body that needs to be intervened in the name of health, quality of life, and well-being; it is also the expression of a series of transformations that influence people’s identity in social and psychological terms. In effect, the word “cancer” and all its “symbolic charge” in diagnostic terms serve as a mechanism for identification and intervention. As stated by Hacking himself, there is a feedback effect between cognition and culture. The knowledge that assists the different participants with their condition in biological, psychological, social, and spiritual terms has modified them in a particular way: it has turned them into “classes of people.”

The group of patients who alluded to determinism or materialism in the SRs of the etiology and treatment of the disease affirms that the cause, and therefore, the treatment, is found in the genes, excluding environmental and behavioral factors; these patients could be identified as subjects who opt for a causal “genetization” of the disease. This concept, coined by the social sciences, refers to the style of thinking applied to the processes and consequences derived from genetic reductionism (Arribas-Ayllon, 2016). It is necessary to avoid genetic reductionism since this generates passive subjects and suppresses social determinants of health (Lippman, 1992). However, Rose (2012) argues that genetization of the disease should generate new ways of subjectification resulting from risk management turning patients into active people who are capable and responsible for their health (Rose, 2012). On the contrary, the narratives of the patients in this study indicate that genetization of cancer takes away the responsibility from them, disassociating the disease from their behavior, social context, and the etiology and therapeutics of the disease; therefore, they invest all their hope in biomedical treatments. Therefore, these representations would not only be descriptive forms but also mechanisms that facilitate intervention in objects (pathology) and patients.

The most common account of patients regarding the etiology and treatment of cancer revolved around socio-anthropological aspects. The disease was attributed to burnout from an excess of activities, transformation into multitasking, pressure to maintain high levels of performance, and self-exploitation to maintain productivity. These etiological factors coincide with the society of fatigue described by Han (2012) and with Alain Ehrenberg’s studies conducted in the nineties; it is characterized by performance as a social imperative, by enterprising subjects who permanently seek to maximize productivity, and by demands leading to self-harm and the manifestation of psychological illnesses. The participants in this study linked these characteristics of the late-modern subject to their condition, a finding that is consistent with the Cancer Barometer survey conducted on 3,120 French citizens which revealed that 73.3% of them consider that the stress of modern life contributes to the occurrence of cancer (Perretti-Watel et al., 2012). A recent meta-analysis showed that work stress is a risk factor for certain types of cancer (Yang et al., 2019). Beyond looking for pathophysiological factors that may explain this relationship or identifying the causal routes that connect cancer with the lifestyles favored by contemporary socio-economic neoliberalization, what is clear in the patients’ accounts is that their condition allowed them to identify a series of social, economic, cultural, and political determinants of cancer that should have a central place in research on this topic and in public policy.

The patients’ narrative alluded to psychogenic aspects in the etiology and treatment of cancer, a concept that emerges both from an internal dialog and from interactions with oncologists and psychologists. This finding coincides with a study that revealed that most people believe that having gone through painful experiences (60.9%), emotional or professional disappointment (49%) and not expressing emotions (38.9%) contribute to the onset of the disease (Perretti-Watel et al., 2012). The dissemination of ideas that attribute the etiology and therapeutics of cancer to psychological aspects may have penetrated popular culture through two main circuits. On the one hand, from the discourses emanating from a sector of the medical establishment itself, such as the case of Snow (1793), who related cancer to mental illness, anxiety, and depression. On the other hand, self-help culture and literature (Illouz, 2010) have co-modified the connection between psychology and cancer through the prolific production of books. In this sense, Hay (2011), considered the driving force behind the personal growth movement, and author of the book “Heal Your Body” and the global bestseller “You Can Heal Your Life,” states in her books that the probable cause of cancer is a deep wound; holding onto resentment; hatred; and believing that everything is useless. The success in disseminating these ideas is because they constitute a powerful narrative framework that appears to offer scientific confirmation of some values and beliefs deeply rooted in different cultures (Illouz, 2010).

Within the psychogenic factors, the notion of proto-disease also emerged to refer to the psychosomatic mechanisms that can be triggered by the fact that the physician informs patients about the risk of getting sick, something that would generate strong negative expectations about their health and thus induce cancer. The notion of proto-disease was coined by the historian of medicine Rosenberg (2003) to problematize the disputes about the limits of disease that emerge in medicine because of the invention of new diagnostic techniques. Rose (2012) argues that the proto-disease generates important changes in lifestyle, including individuals in the world of medicine with examinations, self-examinations, medications, and even creating “pre-patients,” a phenomenon that is very lucrative for the pharmaceutical industry but generates important ethical questions. In this regard, it is important to remember Canguilhem (2001), who, referring to René Leriche, defines health as the silence of the organs and illness as a state of suffering. The link established by patients between the negative thoughts produced by the communication of risks and the origin of their disease reassures the strength with which the positive-thinking movement and the massive self-help industry have spread over health. This situation has important consequences for patients with cancer once they are diagnosed: (i) patients find themselves in an environment where certain rhetorics of happiness and well-being are very strongly extended. Therefore, too much pressure is exerted on them to avoid feeling sadness, although we know that sadness and stress despite adversity constitute a normal part of the process and (ii) happiness becomes an obligation that acquires a totalitarian aspect, insofar as patients are forced to feel in a particular way (Andrade, 2019). This does not imply that one should assume a Schopenhauerian pessimism or that this should be recommended to patients; instead, healthcare professionals should avoid discourses that promote irrational optimism and the false hope that thoughts mystically attract both good and bad outcomes.

The main limitations of this study are the following: a broad clinical spectrum was not analyzed in order to avoid memory bias in the case of patients whose diagnosis occurred several years ago. The change in RS according to the severity of the patient’s type of cancer was also not analyzed in depth. The SRs were based on the discursive material that was divided into codes, this process of categorization in the fundamental theory can imply loss of context of key phrases or significant words. Subsequent studies could remedy these limitations by expanding sampling and applying ethnographic field research techniques. It is also relevant to specify that in this type of study it is not relevant the separation between biomedical undeniable facts and misconceptions-prejudice of the sociocultural domain. Those who wish to allude to facts could complement these findings with a quantitative study.

## 5 Conclusion

Three major conclusions can be extracted from this study. First, the negative representations of cancer, and its association with death persist, despite the significant developments of new technologies, knowledge, and therapies. This finding, added to other researches, suggests that this way of understanding the disease is determined by the convergence of cultural meanings and personal experiences.

Second, there are two etiological representations of cancer that specific forms of coping and managing the disease. Causal representation is connected to the patients’ mechanisms of action, and their disposition in the face of their diagnosis. Within representation oriented toward causal genetization based on a materialistic conception, only biomedical interventions are justified. In this perspective medical norms set by biomedicine prevail over norms of the individual living being, to use Georges Canguilhem’s terminology. The other representation allows for a multicausal perspective in which socioanthropological, and psychological factors highlighted with biological issues. This plurality results in greater regulatory flexibility of patients in the face of medical power, thus opening the way to multiple therapies and disease management strategies.

Third, it is important not to overlook the social and ideological determinants underlying the participants’ accounts. On one hand, patients express almost as a complaint that their illness is the result of an excessive performance. In this case, cancer is expressed as the consequence of a body subjected to excessive productivity whose sources come from a demanding environment, the “interiority” and the self-demanding nature of social discipline. On the other hand, there is a psychogenic predisposition resulting from a medicine of vigilance that is focused on the visibility of etiological risk factors, and environment where the ideology of happiness seems to be a social norm. This double saturation of factors in which the individual is immersed ends up constituting new burdens that are not often visible, and identifiable for caregivers and health professionals.

## Data availability statement

The raw data supporting the conclusions of this article will be made available by the authors, without undue reservation.

## Ethics statement

The studies involving humans were approved by the Bioethics Committee of the Universidad Cooperativa de Colombia record 027-2020 minute 005. This study was conducted according to the guidelines of the Declaration of Helsinki, and Resolution 8430 of the Ministry of Health of Colombia of 1993. The studies were conducted in accordance with the local legislation and institutional requirements. The participants provided their informed consent via audio and/or video to participate in this study.

## Author contributions

LH-G: Conceptualization, Data curation, Formal analysis, Funding acquisition, Investigation, Methodology, Project administration, Resources, Writing – original draft, Writing – review & editing. DE-M: Data curation, Formal analysis, Investigation, Methodology, Validation, Writing – original draft, Writing – review & editing. JC-A: Data curation, Formal analysis, Investigation, Methodology, Validation, Visualization, Writing – original draft, Writing – review & editing.
